# Case Report: MRSA sepsis after pediatric traumatic pseudoaneurysm

**DOI:** 10.3389/fped.2025.1648541

**Published:** 2025-09-10

**Authors:** Xiongfeng Zhang, Hua Zhao, Qingtian Yang

**Affiliations:** Department of Vascular, The Second Clinical College of Guizhou University of Chinese Medicine, Guiyang, Guizhou, China

**Keywords:** femoral artery pseudoaneurysm, pediatric vascular trauma, autologous vein patch angioplasty, MRSA infection, necrotizing fasciitis, closed blunt trauma, therapeutic drug monitoring, sepsis

## Abstract

Pediatric vascular injuries are rare and present unique clinical challenges due to differences in vessel size, injury patterns, and long-term outcomes compared with adults. We report the case of an 11-year-old boy who developed a ruptured and infected superficial femoral artery pseudoaneurysm following closed blunt trauma, which was further complicated by sepsis and necrotizing fasciitis. The patient underwent urgent surgical repair with autologous great saphenous vein patch angioplasty, combined with extensive debridement of necrotic tissue. Postoperatively, methicillin-resistant *Staphylococcus aureus* (MRSA) was identified, necessitating individualized, pharmacokinetically guided vancomycin therapy. This case highlights the importance of early vascular assessment following pediatric blunt trauma, the feasibility of autologous tissue reconstruction in infected arterial injuries, and the critical role of real-time therapeutic drug monitoring in optimizing the management of severe pediatric MRSA infections. This case underscores the need for multidisciplinary coordination and long-term follow-up to optimize outcomes in rare pediatric vascular trauma.

## Introduction

A pseudoaneurysm of the femoral artery following closed blunt trauma is a rare but serious vascular complication in children, associated with a high risk of rupture and significant morbidity (1). Infection can obscure anatomical planes, facilitate bacterial spread, and significantly complicate both surgical and medical management. Most reported cases of superficial femoral artery (SFA) pseudoaneurysm are related to underlying diseases or iatrogenic injuries, and are usually not accompanied by active infection (2, 3). The concurrent occurrence of a pseudoaneurysm and infection in a child is extremely rare, and no specific management guidelines currently exist.

Optimal treatment requires an integrated multidisciplinary approach combining vascular surgical expertise, individualized antimicrobial strategies, and robust infection control measures. Indications for urgent surgical intervention include large hematomas with tissue necrosis or compressive symptoms (e.g., neuropathy, ischemia), rapidly expanding pseudoaneurysms, and infected arterial lesions (4–6). Management choices should be based on lesion size, location, and complexity, while prioritizing limb preservation. Sound preoperative planning, including thorough assessment of both vascular structures and surrounding soft tissues, meticulous intraoperative decision-making, and stringent infection control are critical for achieving favorable long-term outcomes (7). This report presents a successful case, underscoring the importance of coordinated vascular repair and antimicrobial management, and provides insights for the future management of similar cases.

## Case presentation

An 11-year-old boy presented with persistent left thigh pain, swelling, and fever for five days following a closed blunt injury. The incident occurred during a physical education class, when he tripped, fell forward, and struck the anterior aspect of the left thigh. There was no overlying skin laceration and no features suggesting non-accidental trauma. On admission, his temperature was 38.9°C, heart rate 148 bpm, and blood pressure 119/82 mmHg. Physical examination revealed obvious swelling, increased skin temperature, marked tension, and restricted movement of the left lower limb. The mid-thigh circumference was 43 cm (15 cm below the knee) and calf circumference 30 cm (15 cm below the knee), compared with 32 cm and 22.5 cm on the right side. There was no deformity apart from swelling, and radiographs showed no evidence of fracture. Bilateral dorsalis pedis pulses were palpable.

Computed tomography angiography (CTA) of the lower limbs showed significant soft tissue swelling in the left thigh and calf, mild–moderate stenosis of the distal femoral artery, and a localized pseudoaneurysm with surrounding hemorrhage. Laboratory tests showed leukocytosis (WBC: 36.23 × 10^9^/L, neutrophils 94%), thrombocytosis (536 × 10^9^/L), anemia (Hb: 88 g/L), and an elevated C-reactive protein (CRP) of 287.56 mg/L. Ultrasound confirmed rupture of an SFA pseudoaneurysm with surrounding edema and fluid collection. Cardiac ultrasound, chest CT, urinalysis, and autoimmune panel were unremarkable, and no other aneurysms were identified in the vascular structures examined, including the femoral, popliteal, and distal arteries.

He had no prior medical history. The diagnosis prior to surgery was ruptured and infected SFA pseudoaneurysm. Empirical piperacillin–tazobactam and linezolid were initiated, with linezolid selected after multidisciplinary discussion for its superior tissue penetration and efficacy against suspected MRSA and necrotizing fasciitis in pediatric patients. Emergency surgery was planned, with saphenous vein patch repair favored, but ligation reserved as a last resort.

During surgery, a thigh-base incision exposed the ipsilateral great saphenous vein (GSV; 4 mm × 10 cm segment). Proximal SFA control was obtained via a 6F sheath, and DSA localized the pseudoaneurysm. A balloon (4 mm × 40 mm) was used for proximal occlusion. Longitudinal arteriotomy revealed hematoma, necrotic fascia with purulent tracking along the femoral vein and adjacent nerve. Due to severe infection surrounding a 4 mm arterial wall defect, separation of the femoral arteriovenous space was challenging. No direct injury to the femoral vein or nerve was present.

**Figure 1:**
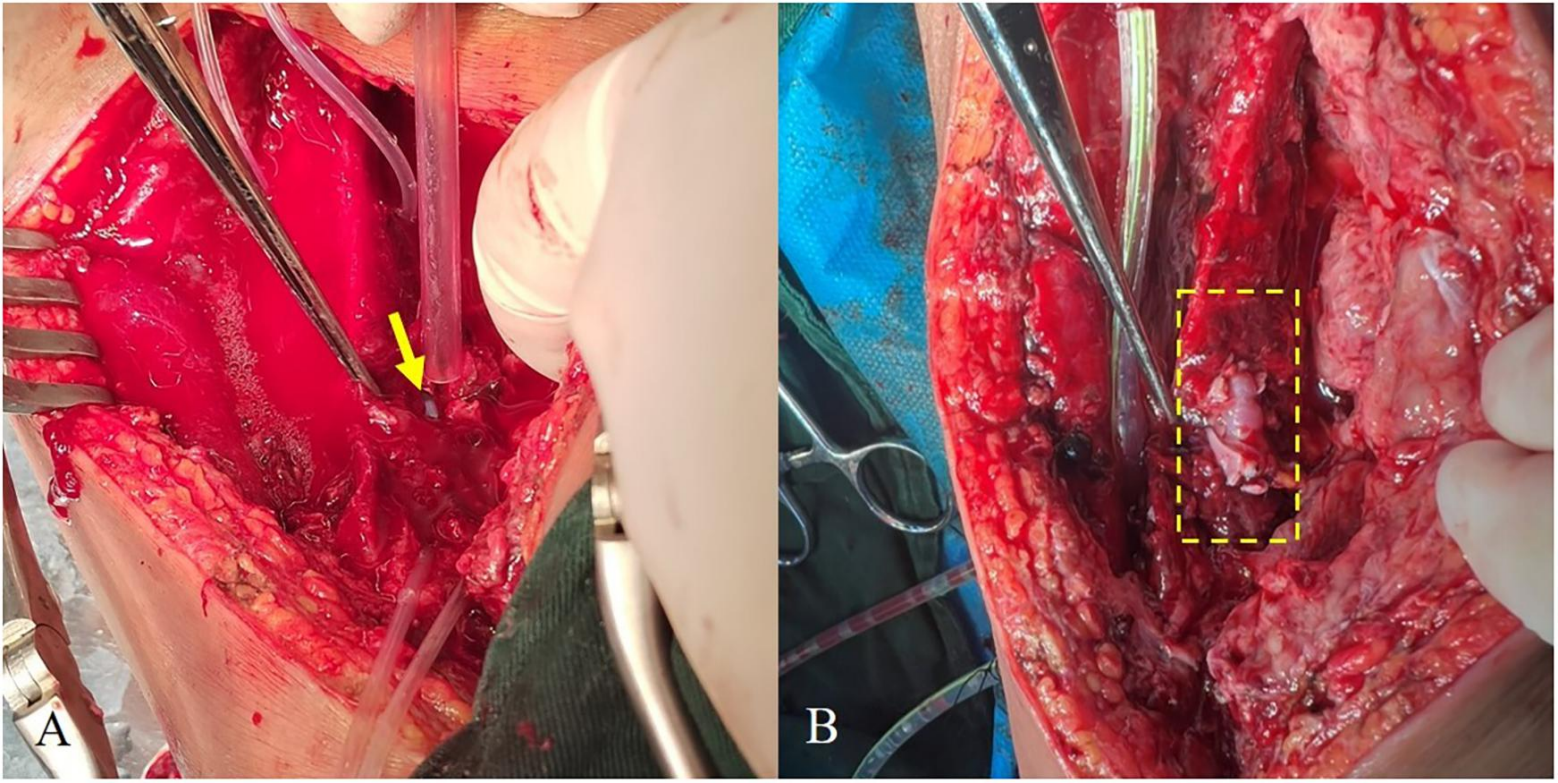
**(A)** Site of arterial rupture (arrows). **(B)** Post-repair vessel, with the repaired segment indicated by a dashed box.

Patch angioplasty was performed using two autologous GSV patches (6-0 Prolene, eversion technique), with distal vessel loops for control. Following debridement and wall reinforcement, restoration of flow showed good patency and no leakage on DSA. The wound was thoroughly irrigated and closed over a negative-pressure drain (Figure 1).

Postoperatively, MRSA was identified from blood and wound cultures. Initial antibiotics were continued, with clinical improvement. Labs normalized (WBC 4.13 × 10^9^/L, Hb 105 g/L, neutrophils 55.7%, platelets 307 × 10^9^/L, CRP 8.91 mg/L). After a week, WBC dropped to 2.17 × 10^9^/L; hematologic toxicity from antibiotics was suspected, so therapy was switched to vancomycin.

Initial vancomycin dosing (600 mg q6h, 15 mg/kg) was subtherapeutic [trough 8.53 µg/ml, area under the concentration–time curve over 24 h/minimum inhibitory concentration ratio (AUC_24_/MIC) 232]. On day 7, fever recurred and CT revealed a 3.2 × 2.7 cm perigraft fluid collection. Surgical debridement and vacuum-assisted surgical drainage (VSD) were performed. Vancomycin was escalated to 800 mg q6 h (80 mg/kg/d), achieving target trough (14.61–18.52 µg/ml) and AUC₍_24_₎/MIC (421–477) within 72 h. Recovery was uneventful. VSD was removed after one week, the wound was closed, and intravenous vancomycin therapy was continued at home for 4 weeks after hospital discharge.

The patient demonstrated steady recovery. At the 2-month follow-up, mid-thigh and calf circumferences were comparable to those of the contralateral limb. At 6 months, CTA confirmed complete arterial healing with sustained patency. By 12 months, no growth-related discrepancies in limb circumference or anatomy were detected. Ongoing long-term surveillance is planned to identify any delayed complications or growth disturbances.

**Figure 2:**
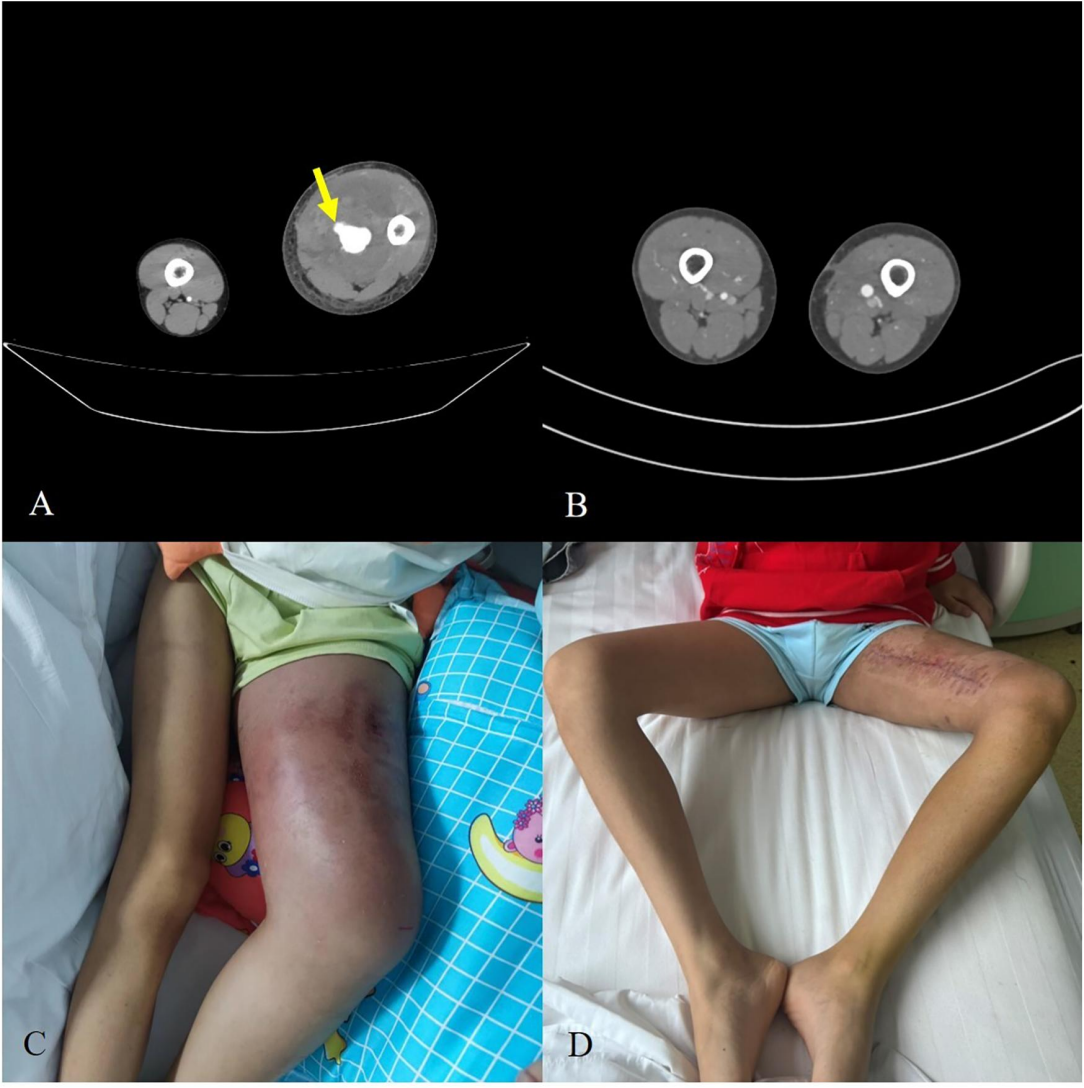
**(A)** Preoperative CTA demonstrating contrast extravasation from the pseudoaneurysm (arrows). **(B)** Postoperative CTA showing restored vessel patency. **(C)** Clinical photograph of preoperative thigh swelling. **(D)** Postoperative recovery with significant reduction of swelling and healed incision.

The perioperative findings are illustrated in Figure 2. Preoperative CTA (Figure 2A) revealed active contrast extravasation, indicating ongoing arterial bleeding from the pseudoaneurysm. After endovascular repair, follow-up CTA (Figure 2B) demonstrated restoration of vessel patency. Clinical photographs before surgery (Figure 2C) showed significant thigh swelling, while postoperative images (Figure 2D) revealed substantial reduction of swelling and complete wound healing, confirming the effectiveness of our management.

## Discussion and conclusions

Pediatric SFA pseudoaneurysms are extremely rare, particularly in the absence of penetrating trauma, fractures, or underlying connective tissue disorders (8). Most reported cases are associated with high-energy mechanisms, such as open injuries or fractures, which makes this case of a healthy child with a closed blunt trauma unique and noteworthy. In this patient, the pseudoaneurysm likely resulted from focal arterial wall disruption due to blunt trauma, despite the absence of bone injury or penetration (9). Unlike classic Morel-Lavallée lesions or crush injuries, the delayed onset of swelling and fever indicated that vascular injury preceded the development of infection (10). Research has shown that damaged muscle tissue creates an ideal environment for bacterial colonization. When combined with a ruptured arterial wall, this facilitates hematogenous spread, potentially leading to systemic sepsis if not promptly recognized and treated (11–14). This case emphasizes the need for early vascular assessment following pediatric blunt trauma, even without external wounds or fractures.

The management of infected pseudoaneurysm in children is highly challenging and must be tailored to both the extent of infection and anatomical factors (15–18). While endovascular or minimally invasive approaches (such as ultrasound-guided thrombin injection) have been reported as effective for non-infected pseudoaneurysms, their role becomes limited in situations complicated by active sepsis or extensive tissue necrosis. In such cases, endovascular repair may serve only as a temporary or bridging option, whereas timely open surgical intervention is generally regarded as the safer and more durable solution (19, 20).

Although simple arterial ligation has been advocated in adult series as a rapid means to control infection and prevent further complications (21, 22), pediatric experience is limited, and this strategy carries a substantial risk of limb ischemia and long-term functional impairment in growing children. Several reports and reviews recommend that, whenever feasible, revascularization with autologous tissue should be prioritized to preserve limb growth and function (20, 23, 24).

In our patient, extensive local infection and the difficulty in separating involved arterial and venous structures rendered interposition grafting with a long segment of saphenous vein impractical and potentially hazardous, given the high risk of graft reinfection. We therefore selected a local autologous GSV patch angioplasty, reinforced with double patching and muscle wrapping, to minimize foreign material, preserve native tissue, and reduce the risk of reinfection or re-rupture. This approach is supported by recent pediatric vascular literature, which favors autologous repair and interrupted suturing in small vessels to promote long-term patency and limb growth (25). Comprehensive debridement and reliable drainage were equally crucial for infection control.

Empiric antibiotic therapy for pediatric MRSA sepsis should be initiated promptly in accordance with Surviving Sepsis Campaign recommendations (26), and subsequently adjusted based on culture results and pharmacokinetic/pharmacodynamic (PK/PD) monitoring. In our case, the specific details of vancomycin dosing, trough levels, and AUC_24_/MIC monitoring were omitted here to maintain focus on the surgical management; however, antibiotic optimization played a crucial role in controlling infection, particularly when initial therapy proved suboptimal (27–29). This experience reinforces the importance of individualized, PK-guided antibiotic dosing as a standard of care in severe pediatric MRSA infections, especially in complex surgical patients.

This case underscores several key clinical lessons: 1. early suspicion and vascular imaging; 2. individualized operative strategies with autologous reconstruction and infection control; 3. dose optimization of antibiotic therapy guided by monitoring. Multidisciplinary cooperation and careful long-term follow-up are necessary to optimize outcomes and monitor for late complications, such as patch degeneration or limb dysfunction. Although our follow-up was limited to 6 months, continued monitoring is underway to assess durability and growth-related sequelae. Studies have demonstrated that pediatric vascular lesions and reconstructions may result in growth disturbances—including limb length discrepancy and impaired bone development—particularly when vascular perfusion is not fully restored. Accordingly, long-term follow-up and growth-accommodating surgical strategies are essential to mitigate these risks (30, 31).

## Data Availability

The raw data supporting the conclusions of this article will be made available by the authors, without undue reservation.
